# Propylene Glycol Alleviates Oxidative Stress and Enhances Immunity in Ketotic Cows through Modulating Amino Acid and Lipid Metabolism

**DOI:** 10.3390/antiox13091146

**Published:** 2024-09-23

**Authors:** Jian Tan, Huiying Zhao, Liuxue Li, Ying Wang, Yucong Pan, Luoyun Fang, Yuchao Zhao, Linshu Jiang

**Affiliations:** Beijing Key Laboratory of Dairy Cow Nutrition, College of Animal Science and Technology, Beijing University of Agriculture, Beijing 102206, China; 202230312013@bua.edu.cn (J.T.); 202130312010@bua.edu.cn (H.Z.); 202130312014@bua.edu.cn (L.L.); 202230321112@bua.edu.cn (Y.W.); 93219702@bua.edu.cn (Y.P.); fangly@bua.edu.cn (L.F.)

**Keywords:** dairy cows, ketosis, propylene glycol, lipid metabolism

## Abstract

This study investigates the impact of propylene glycol (PRG) on ketotic cows, focusing on alleviating oxidative stress and enhancing immunity through modulating amino acid and lipid metabolism. Ketosis, a prevalent metabolic disease in dairy cows, negatively affects productivity and health. PRG, known for its gluconeogenic properties, was administered to cows with ketosis daily for three days and compared to an untreated group. Serum samples were taken to measure the biochemical parameters, and metabolomic and lipidomic analyses were performed with ultra-high-performance liquid chromatography–mass spectrometry. The results showed significant reductions in serum non-esterified fatty acids, beta-hydroxybutyrate, and C-reactive protein levels, alongside increased glucose, anti-inflammatory factor interleukin-10, superoxide dismutase, and glutathione peroxidase activities. Metabolomic and lipidomic analyses revealed significant alterations, including increased levels of glucogenic amino acids like glutamate and proline, and decreased levels of ceramide species. A pathway analysis indicated that PRG affects multiple metabolic pathways, including alanine, aspartate, glutamate metabolism, and sphingolipid metabolism. These findings suggest that PRG not only mitigates oxidative stress, but also enhances immune function by restoring metabolic homeostasis. This study provides valuable insights into the biochemical mechanisms underlying PRG’s therapeutic effects, offering potential strategies for the effective management and treatment of ketosis in dairy cows.

## 1. Introduction

With the continuous global rise in demand for dairy products, the incidence of metabolic diseases in dairy cattle has significantly increased, adversely affecting their productivity and overall welfare [1]. The periparturient period, defined as three weeks before to three weeks after parturition, is crucial for the health and economic output of dairy cows. This period is marked by significant physiological, nutritional, metabolic, and immunological changes [2]. Successful adaptation to the periparturient period and the accompanying negative energy balance (NEB) can ensure a healthy and productive lactation phase. Conversely, poor adaptation can lead to various issues, including clinical diseases and reduced milk production [3]. Ketosis, one of the most prevalent diseases in dairy cows, often occurs alongside conditions such as lameness, displaced abomasum, metritis, mastitis, retained placenta, and hypocalcemia [4]. It is also recognized as a significant risk factor for other diseases [3]. Cows suffering from ketosis are at an increased risk of developing additional metabolic disorders and being culled, and they also experience decreased milk yield and reproductive performance [5,6].

Subclinical ketosis (SCK) is a pathological state characterized by the accumulation of ketone bodies in the blood, urine, and milk without evident clinical symptoms. This condition poses potential risks due to its insidious nature and may progress to clinical ketosis (CK). Clinical ketosis is marked by significantly elevated ketone body concentrations in body fluids, accompanied by reduced appetite, decreased milk production, rapid weight loss, and dry feces [7]. During the periparturient period, cows with ketosis experience disrupted amino acid metabolism, particularly with decreased levels of ketogenic and glucogenic amino acids such as asparagine, histidine, methionine, serine, alanine, leucine, and lysine [8,9]. The NEB associated with ketosis reduces the availability of amino acids for gluconeogenesis. Concurrently, the high energy demands for milk production post-calving increase fatty acid oxidation, replacing glucose as the primary oxidative fuel. In NEB conditions, hypoglycemia triggers fat mobilization, leading to the accumulation of non-esterified fatty acids (NEFAs) in the liver, the production of advanced glycation end products, and increased oxidative stress, resulting in various metabolic dysfunctions. Therefore, understanding the role of amino acids in the development of ketosis is crucial for improving the metabolic health of ketotic cows.

Oxidative stress plays a central role in the pathogenesis of ketosis, arising from an imbalance between the production of reactive oxygen species (ROS) and the body’s antioxidant defense systems. This imbalance leads to cellular damage, increased inflammation, and impaired immune function [10]. Therefore, mitigating oxidative stress is crucial for improving the health and productivity of cows affected by ketosis. Numerous studies have reported disturbances in lipid metabolism in ketotic cows, including abnormal hepatic triglyceride (TG) deposition and decreased cholesterol levels [11,12,13]. Even weeks before clinical diagnosis, affected cows exhibit activated innate immunity and altered carbohydrate and lipid metabolic pathways [11]. Recent research by Zhao et al. [14] further reveals that SCK disrupts immunometabolic homeostasis and induces oxidative stress in adipose tissue, emphasizing the critical role of sphingolipid metabolism in this process. Specifically, ceramide accumulation is linked to heightened inflammation and oxidative stress, exacerbating insulin resistance and metabolic dysregulation [15]. These findings underscore the urgent need for targeted interventions, such as propylene glycol, to modulate lipid metabolism, alleviate oxidative stress, and restore metabolic balance in ketotic cows.

Since 1950, propylene glycol (PRG) has been used to treat ketosis due to its gluconeogenic properties [16,17]. PRG is hypothesized to work by providing an alternative source of glucose through the process of gluconeogenesis, thereby reducing the reliance on lipid mobilization and preventing the excessive accumulation of ketone bodies [13]. Additionally, PRG has been suggested to enhance insulin sensitivity and reduce hepatic gluconeogenesis from amino acids, sparing them for protein synthesis and other metabolic functions [14]. Research by McArt et al. [18,19] further confirmed that oral drenching with PRG effectively reverses hyperketonemia, reduces the risk of abomasum displacement, lowers early lactation-culling rates, and increases milk production. These findings suggest that PRG drenching could be an efficient intervention for physiological imbalance. Numerous studies have investigated the effects of different PRG dosages, stages of lactation, routes of administration, and treatment durations, as well as its combination with other antilipolytic agents [20,21,22]. However, no research to date has specifically examined the impact of PRG on lipid metabolism in ketotic cows.

Metabolomic and lipidomic analyses offer comprehensive approaches to understand the biochemical alterations associated with ketosis and the therapeutic effects of PRG. These techniques enable the identification and quantification of metabolites and lipids, providing insights into the metabolic pathways and molecular mechanisms involved [23]. Despite the growing body of research, a gap exists in understanding the mechanistic effects of PRG on lipid metabolism and its broader implications on the immunometabolic health of ketotic cows. This study aims to address this gap by investigating the effects of PRG on systemic metabolic status and metabolite profiles in cows with ketosis using metabolomic and lipidomic approaches. We hypothesize that PRG drenching alleviates oxidative stress and enhances immune function by modulating amino acid and lipid metabolism, contributing to the recovery from ketosis. Understanding these effects at the molecular level could inform better management and therapeutic strategies for ketosis in dairy cows.

## 2. Materials and Methods

### 2.1. Experimental Design and Animal Management

All animal research was performed under a protocol approved by the Animal Care Committee of Beijing University of Agriculture, with approval number BUA2022055.

During the first three weeks of lactation, all multiparous peripartum cows (*n* = 62) on the farm were monitored daily for ketosis. Blood samples were collected every morning before feeding during the first three weeks of lactation. For sample collection, cows were restrained in headlocks, and approximately 6 mL of blood was drawn from the coccygeal vessels using an 18-gauge × 2.54 cm needle and a 6 mL syringe. Immediately after collection, β-hydroxybutyric acid (BHBA) levels were measured using a Precision Xtra meter (Abbott Laboratories) and corresponding blood ketone test strips. Following the ketone measurement (which appears 10 s after blood application), glucose levels were tested and displayed within 5 s. The Precision Xtra method for glucose determination has been validated in cattle, with a sensitivity of 76.2% and specificity of 92.7% at a 2.2 mmol/L cutoff [24]. Only cows exhibiting hyperketonemia (BHBA > 1.2 mmol/L) were included in the study. Cows were excluded if a trained veterinarian diagnosed them with any concurrent disease at enrollment, such as displaced abomasum, retained placenta, lameness, metritis, pneumonia, or clinical mastitis. Additionally, animals developing any of these conditions within 14 days post-enrollment were subsequently removed from the trial.

The cows were randomly assigned to two groups: (1) the PRG group (*n* = 6), which received an oral dose of 300 mL of propylene glycol daily for three consecutive days, and (2) the control group (CON, n = 6), which did not receive any special treatment. For the PRG group, administration began on the day their BHBA levels reached or exceeded 1.2 mmol/L, with 300 mL of propylene glycol provided daily for three days by Sino Biopharmaceutical Co., Ltd., Chongqing, China. The 300 mL dose was chosen as it is a common standard dose used on farms, approximately equivalent to 310 g of propylene glycol. During the experiment, the cows were housed in individual stalls with sawdust bedding and feed bins until the end of the study period (21 days in milk, DIM). The cows were fed a total mixed ration (TMR) prepared daily at 9:00 AM and fed at 9:30 AM and 3:00 PM, aiming for a 10% refusal rate. Details on the TMR’s ingredients and chemical composition are provided in Table S1. The cows were kept in a freestall barn, each with a sand-bedded individual stall, and assigned to individual feeding gates (Roughage Intake Control System). Basic information about the cows used in the study is presented in Table S2. On the day of ketosis diagnosis, there were no significant differences between the two groups in terms of parity, body weight (BW), body condition score (BCS), milk yield, dry matter intake (DMI), BHBA levels, or DIM.

### 2.2. Blood Collection and Serum Biochemical Parameters Determination

Blood samples for the determination of serum biochemical parameters were collected from all enrolled cows the day before PRG administration (i.e., the day of ketosis diagnosis) and after three consecutive days of PRG drenching. Sterile polyethylene terephthalate tubes were used to collect serum (BD Vacutainer TM for trace element testing with serum clot activator). After centrifugation, the serum samples were stored as follows: one aliquot at −20 °C for biochemical analysis and two aliquots at −80 °C for metabolomic and lipidomic analyses. Serum superoxide dismutase (SOD), glutathione peroxidase (GSH-Px), NEFA, BHBA, glucose (GLU), TG, and total cholesterol (TC) were measured using commercial colorimetric assay kits (Beijing Solarbio Science & Technology Co., Ltd., Beijing, China). Serum concentrations of C-reactive protein (CRP), IL-1β, IL-2, IL-6, IL-10, TNF-α, insulin, leptin, and adiponectin were measured using commercial bovine-specific ELISA kits according to the manufacturer’s instructions (Nanjing Jiancheng Bioengineering Institute, Nanjing, China). It is important to note that the coefficients of variation for all assays, both within and between groups, were maintained below 10%, ensuring the accuracy and reliability of the data. To further evaluate insulin sensitivity, we calculated the revised quantitative insulin sensitivity check index (RQUICKI) using the formula RQUICKI = 1/[log(serum glucose mg/dL) + log(serum insulin μU/mL) + log(serum NEFA mmol/L)], as described by Holtenius and Holtenius [25]. A lower RQUICKI value indicates reduced insulin sensitivity.

### 2.3. Serum Metabolomics Analysis

After thawing the serum samples at room temperature, metabolites were extracted using the methanol protein precipitation method described by Zhao et al. [15]. The extracts were redissolved in 200 µL of water and stored at −80 °C for subsequent metabolomic analysis. The metabolites were analyzed using ultra-high-performance liquid chromatography–mass spectrometry (UHPLC-MS) with a Vanquish Horizon system and a Q-Exactive HF-X mass spectrometer (Thermo Scientific, San Jose, CA, USA), equipped with a 2.1 mm × 100 mm Acuity BEH 1.7 µm C18 column (Waters, Milford, MA, USA). The gradient elution was performed using a solvent system comprising 0.1% acetic acid in water (solvent A) and 100% acetonitrile (solvent B). The elution protocol was as follows: 0 to 8 min, 5% to 60% B; 8 to 18 min, 60% to 97% B; 18 to 21 min, 97% B; 21 to 21.1 min, 97% to 5% B; 21.1 to 25 min, 5% B. The flow rate was set at 0.4 mL/min, and the injection volume was 2 µL. Quality control (QC) samples were included in the analytical sequence after every eight samples to ensure data reliability.

Metabolites were identified using the Kyoto Encyclopedia of Genes and Genomes (KEGG), Human Metabolome Database (HMDB), and an in-house database. Peaks with a relative standard deviation (RSD) exceeding 30% in the QC samples were excluded. The processed data were then formatted into CSV files for further analysis.

### 2.4. Serum Lipidomics Analysis

Following the methodology described by Xia et al. [26], we extracted serum lipids. The dried residues were reconstituted in a solvent mixture of acetonitrile, isopropanol, chloroform, and water in a 35:35:20:10 volume ratio, and then injected into the UPLC-MS system for analysis. Lipid analysis was performed on an Acquity UPLC BEH C18 column (50 mm × 2.1 mm; 2.5 µm) using a Vanquish ultra-high-performance liquid chromatography system (Thermo Fisher Scientific, San Jose, CA, USA) coupled with a Q Exactive hybrid quadrupole-Orbitrap mass spectrometer (Thermo Fisher Scientific, San Jose, CA, USA). The mobile phase consisted of 1% 1 M NH₄OAc and 0.1% acetic acid in water (Buffer A), and acetonitrile/isopropanol (7:3, UPLC grade, BioSolve, Valkenswaard, The Netherlands) supplemented with 1 M NH_4_OAc and 0.1% acetic acid (Buffer B). The gradient elution protocol was as follows: initial 1 min at 45% A, and then a linear gradient to 35% A over 3 min, followed by a further linear gradient to 11% A over 8 min, and finally down to 1% A over 3 min. The column was washed for 3 min with 1% A, then re-equilibrated to 45% A for 4 min, making the total run time 22 min. The injection volume was 2 μL for both positive and negative ion modes. The mass spectrometry parameters were set according to our previous study [27], with mass calibration performed after every 8 injections using the instrument’s built-in calibration system.

The LC-MS system was controlled and the data processed using Xcalibur 2.2 SP1.48 software (Thermo Fisher Scientific). Lipid species were identified and quantified using Lipidsearch software (version 4.1.16; Thermo Fisher Scientific, Waltham, MA, USA). Lipids with a relative standard deviation exceeding 30% in quality control samples were excluded, and all sample data were normalized by sum.

### 2.5. Statistical Analysis

The normality of residues was assessed with the Shapiro–Wilk test using the univariate procedure in SAS (version 9.4; SAS Institute Inc., Cary, NC, USA). The analyses of serum variables were conducted with the MIXED mode of SAS, with treatment as the fixed effect and block as the random effect. Serum variables determined on the day of ketosis diagnosis were used as covariate in the model. The Kenward–Roger degrees of freedom method was used to determine denominator degrees of freedom. Statistical significance was considered at *p* ≤ 0.05, while tendencies were considered when 0.05 ≤ *p* < 0.10. The results were reported as least-square means ± standard error of the mean.

Metabolite and lipid data were imported into the SIMCA-P software (version 13.0; Umetrics, Umeå, Sweden) for analysis. The preprocessing steps included log transformation, median normalization, and autoscaling. These steps aimed to eliminate undesirable inter-sample variations, ensure data comparability, stabilize variance, and assign equal weight to all metabolites in subsequent analyses. Multivariate analyses included principal component analysis (PCA) and orthogonal partial least-squares discriminant analysis (OPLS-DA). Differential metabolites and lipids were identified based on the variable importance in the projection (VIP) value from the OPLS-DA model, with criteria of VIP >1 and a significance level of *p* < 0.05. The *p*-values were adjusted for multiple comparisons using the Benjamini–Hochberg (BH) method to control the false discovery rate. This adjustment involved comparing each *p*-value with a predetermined threshold. A hierarchical clustering heatmap was then generated to visualize and identify trends in significant metabolites between groups. Finally, metabolic pathway analysis was conducted using Metaboanalyst 6.0 (https://www.metaboanalyst.ca/ (accessed on 7 July 2024)).

## 3. Results

### 3.1. Serum Metabolic Status Parameters

Serum glucose and lipid metabolism biomarkers showed no significant differences between the CON and PRG groups on the day before propylene glycol administration (Table S3). The effects of PRG drenching on serum glucose and lipid metabolism biomarkers are presented in Table 1. Serum NEFA (*p* = 0.005) and BHBA (*p* = 0.010) were significantly lower in the PRG group than the CON, where the glucose concentration was higher for PGR cows than CON cows (*p* = 0.019). Oral PRG drenching did not affect (*p* > 0.10) serum concentrations of insulin, TG, or adiponectin. Compared with the CON group, PRG drenching significantly reduced the concentration of leptin (*p* = 0.048). We also observed a greater value of RQUICKI in PRG cows than in CON cows (*p* = 0.004).

The serum antioxidant and inflammation indicators were also not significantly different between the CON and PRG groups on the day before propylene glycol administration (Table S4). The effects of RPG on serum antioxidant enzymes, endotoxin, and inflammation factors are reported in Table 2. Compared with CON, the oral drench of PRG increased the activities of SOD (*p* = 0.004) and GSH-Px (*p* < 0.001). The serum LPS content was also lower for the PRG group than the CON group (*p* = 0.019). Ketotic cows administrated with PRG had lower serum CRP than CON cows (*p* = 0.028). Serum IL-10 concentration was higher for PRG cows relative to CON cows (*p* = 0.048). PRG drenching did not affect the concentrations of IL-1β or IL-2 (*p* > 0.010). A tendency (*p* = 0.098) for lower IL-6 concentration was observed in PRG cows relative to CON cows.

### 3.2. Serum Metabolomics Profiling

**Figure 1:**
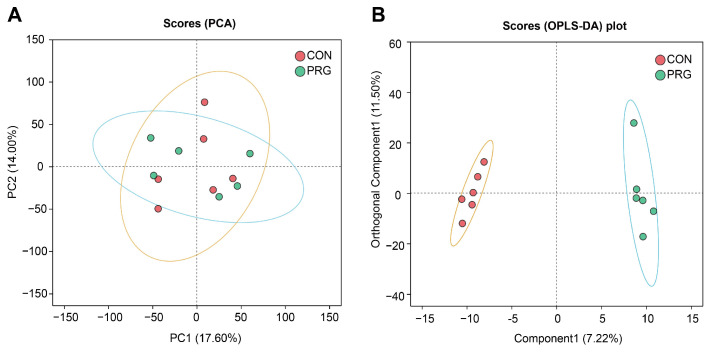
Multivariate analysis score plots of serum metabolites. (**A**) Score plot of the principal component analysis (PCA). (**B**) Score plot of orthogonal partial least-squares discriminant analysis (OPLS-DA). CON, ketosis cows without treatment; PRG, ketosis cows with propylene glycol drenching.

**Figure 2:**
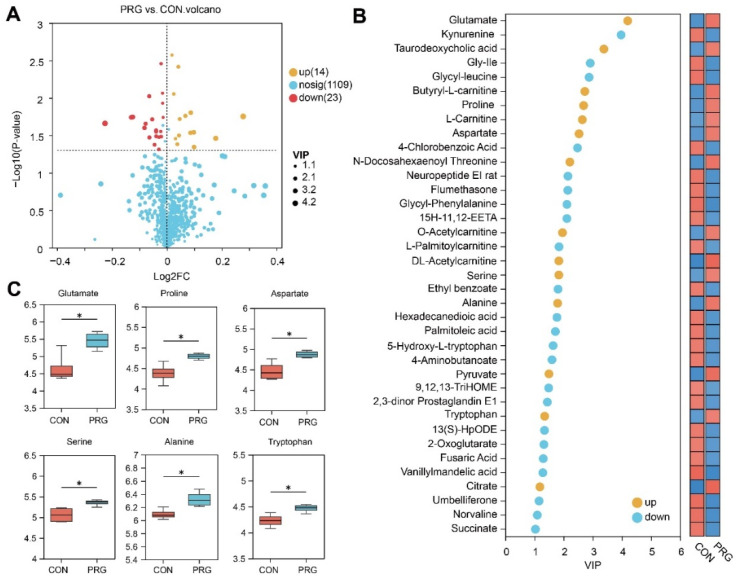
Serum metabolome analysis. (**A**) Volcano plot of serum metabolites. (**B**) The VIP loading plot represents the variable importance in projection (VIP) of each differential metabolite: red represents a high concentration and blue represents a low concentration. (**C**) Examples of boxplots of differential amino acid metabolites. CON, ketosis cows without treatment; PRG, ketosis cows with propylene glycol drenching; VIP, variable importance in projection. * *p* < 0.05.

After removing metabolic redundancies and duplicates, a total of 1146 metabolites were identified using LC-MS. Initially, we conducted a PCA analysis, an unsupervised method, to investigate whether different groups showed clustering patterns. However, the PCA results indicated that there was no clear clustering among the groups (as shown in Figure 1A). Therefore, we proceeded with OPLS-DA to maximize the separation between the groups. As shown in Figure 1B, the serum metabolomes of the CON and PRG were clearly separated following OPLS-DA analysis. Based on a VIP score greater than 1 and a *p*-value less than 0.05, we identified a total of 37 differential metabolites in both positive and negative ion modes (Figure 2A). Among these, the levels of 14 metabolites (e.g., glutamate, taurodeoxycholic acid, butyryl-L-carnitine, proline, L-carnitine, aspartate) exhibited an elevation, whereas the levels of the other 23 metabolites (e.g., kynurenine, gly-Ile, glycyl-leucine, 4-chlorobenzoic acid) demonstrated a reduction in the PRG group compared with the CON group (Figure 2B and Table S5). In particular, several amnio acids (glutamate, proline, aspartate, serine, alanine, tryptophan) were increased in ketotic cows administrated with PRG (Figure 2C).

### 3.3. Serum Lipidome Profiling

**Figure 3:**
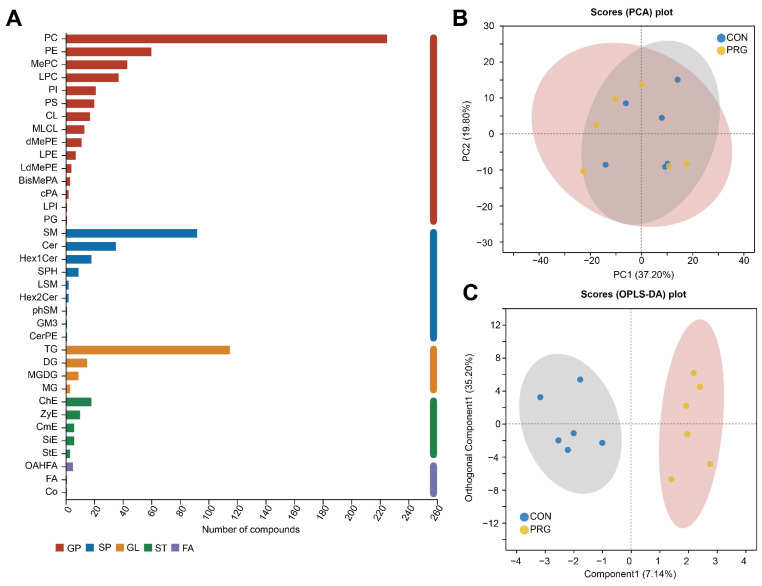
Overview of serum lipidomic analysis. (**A**) Number of lipid species within each identified lipid class. (**B**) Principal component analysis (PCA) of mouse serum lipidome. (**C**) Score plot of orthogonal partial least-squares discriminant analysis (OPLS-DA). CON, ketosis cows without treatment; PRG, ketosis cows with propylene glycol drenching.

In the first part of the study, we characterized the untargeted lipidome of cows’ serum, identifying 769 lipid species. These lipid species from 36 different lipid classes, and phosphatidylcholines (PC, 225 species), TG (115 species), sphingomyelins (SM, 92 species), and phosphatidylethanolamines (PE, 60 species), were the most abundant lipid classes (Figure 3A). We initially employed a PCA, an unsupervised method, to provide an overview and identify potential differences between groups. However, the results showed that the groups did not cluster distinctly (Figure 3B). To further investigate the differences between groups, we then applied OPLS-DA to maximize the separation between them (Figure 3C). The OPLS-DA provided clear evidence of the potential effect of PRG drenching on the lipidomic profile of serum samples of ketotic cows.

**Figure 4:**
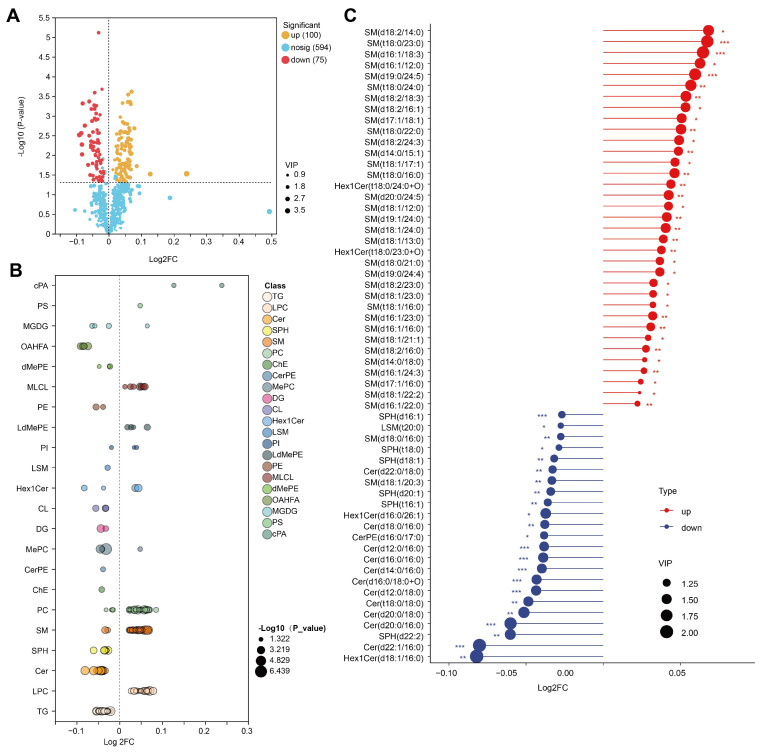
Identification of serum differential lipid species. (**A**) Volcano plot showing differential lipid metabolites. (**B**) Differential lipid species within each identified lipid class. (**C**) Differential sphingolipid species. CON, ketosis cows without treatment; PRG, ketosis cows with propylene glycol drenching; VIP, variable importance in projection. * *p* < 0.05; ** *p* < 0.01, *** *p* < 0.001.

The relative concentrations of 175 lipid species were altered by PRG relative to CON (Table S6). The relative concentrations of 100 species, including cPA (18:2), cPA (16:0), PC (18:3/18:2), and LdMePE (18:2) were increased in PRG cows (Figure 4A). Conversely, 75 species, including OAHFA (18:1/18:0), Hex1Cer (d18:1/16:0), and Cer (d22:1/16:0), were lower in the PRG group relative to the CON group. We created a bubble chart to visually illustrate the differences between the CON and PRG groups across various lipid classes. A reduction in (O-acyl)-1-hydroxy fatty acid (OAHFA), sphingosine (SPH), Cer, and TG in serum samples was shown in cows administrated with PRG (Figure 4B). However, we observed a significantly enhanced level of monolysocardiolipin (MLCL), PC, sphingomyelin (SM), and lysophosphatidylcholine (LPC) in the serum of PRG cows compared with CON cows. In this study, we focused on the sphingolipid metabolism. Among differentially altered lipids, 6 species of SPH, 2 species of SM, 1 species of lysosphingomyelin (LSM), 2 species of monohexosylceramide (Hex1Cer), 1 species of ceramide phosphoethanolamine (CerPE), and 11 species of Cer were lower, but 33 species of SM and 2 species of Hex1Cer were greater in the PRG group (Figure 4C).

### 3.4. Pathway Analysis and Association Analysis

**Figure 5:**
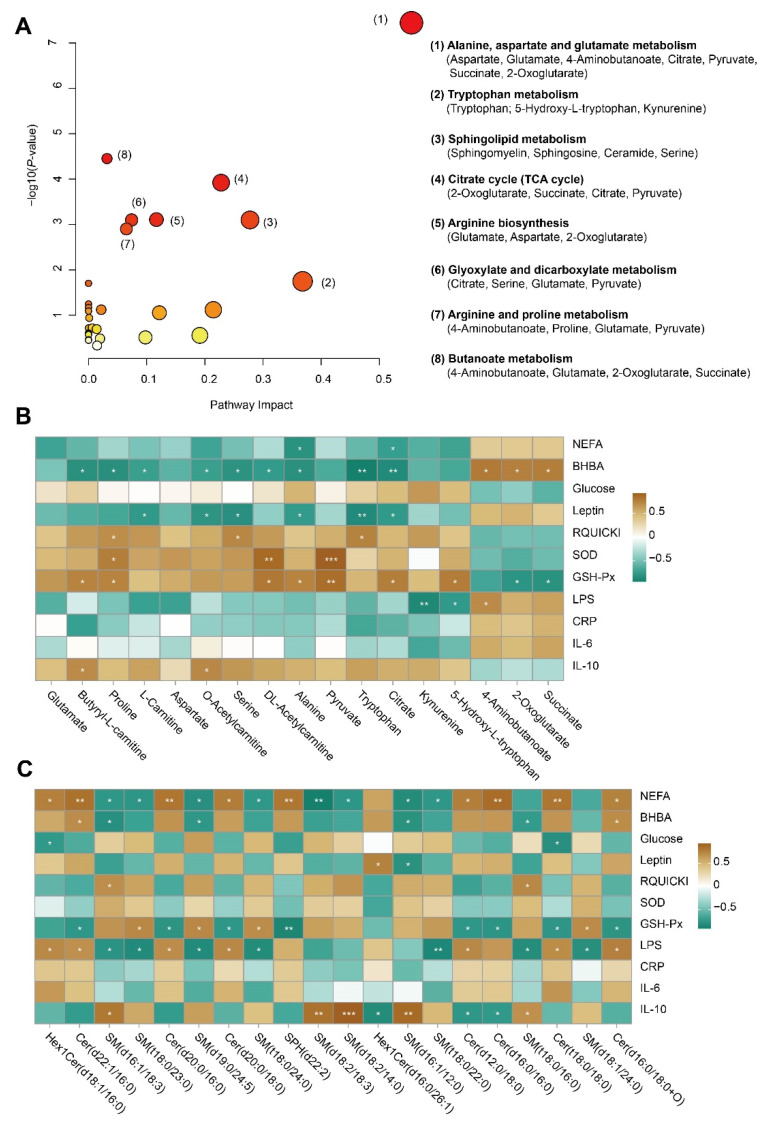
Metabolic pathway analysis and association analysis. (**A**) Pathway analysis result for differential metabolites and lipids between PRG versus CON. (**B**) Association analysis between differential serum biochemical parameters and differential metabolites related to amino acid and energy metabolism. (**C**) Association analysis between differential serum biochemical parameters and differential sphingolipid species (top 20 of VIP). * *p* < 0.05, ** *p* < 0.01, *** *p* < 0.001. NEFA, non-esterified fatty acid; BHBA, β-hydroxybutyric acid; RQUICKI, revised quantitative insulin sensitivity check index; SOD, superoxide dismutase; GSH-Px, glutathione peroxidase; LPS, lipopolysaccharide; CRP, C-reactive protein; IL, interleukin; VIP, variable importance in projection.

Pathway analysis of differential metabolites and lipid species was performed using MetaboAnalyst; the metabolome view is shown in Figure 5A. The results revealed “alanine, aspartate and glutamate metabolism”, “tryptophan metabolism”, “sphingolipid metabolism”, “citrate cycle (TCA cycle)”, “arginine biosynthesis”, “glyoxylate and dicarboxylate metabolism”, “arginine and proline metabolism”, and “butanoate metabolism” as some of the most impacted pathways. Together, these metabolites and lipids mainly involved energy metabolism, amino acid metabolism, and lipid metabolism. Pearson’s correlation analysis was employed to determine the correlations among differentially metabolites related to energy and amino acid metabolism and serum biochemical parameters. We observed that serum BHBA and leptin were negatively correlated with L-carnitine, O-acetylcarnitine, serine, alanine, tryptophan, and citrate (Figure 5B). Serum SOD was positively correlated with proline, DL-acetylcarnitine, and pyruvate. Next, we analyzed the correlations between serum biochemical indices and differential serum sphingolipid species. We noted that serum NEFA, GSH-Px, LPS, and IL-10 were correlated with some sphingolipid species (Figure 5C).

## 4. Discussion

In a recent study, we found that serum NEFA, BHBA, and proinflammatory factor concentrations were increased, and the antioxidant enzyme (SOD and GSH-Px) activities and systemic insulin sensitivity were decreased in cows with ketosis compared with healthy cows [14]. When dairy cows have NEB, lipid mobilization increases, with increased production of NEFA and BHBA. Cows with ketosis are challenged by metabolic stress [14,28]. Furthermore, a marked change in glucose, adiponectin, proinflammatory factors, SOD, and GSH-Px levels indicated that ketotic cows also experience glucose and lipid metabolism disorders, inflammation, and oxidative stress [14]. Here, we found that serum NEFA, BHBA, LPS, and CRP concentrations decreased, and the SOD and GSH-PX activity, glucose, and anti-inflammatory factor IL-10 increased in the PRG group compared with the CON group. Therefore, these results indicated that this stressed state could be dramatically reduced following PRG drenching.

Our aim was to increase glucose availability in ketotic dairy cows by administering propylene glycol and to investigate its effects on metabolic function by screening the serum metabolome. Unlike targeted metabolomics, our untargeted metabolomics analysis based on LC-MS/MS utilizes a data-driven approach to detect and quantify metabolites without predefined chemical targets [29]. This method enables the comprehensive profiling of metabolites, providing deeper insights into the functioning of metabolic pathways. Through the application of this untargeted metabolomics approach, we observed changes in the abundance of several metabolites in both serum and milk, indicating potential interactions related to glucose availability and metabolic pathway activities.

Ketotic cows treated with PRG exhibited significant changes in their metabolic pathways, specifically involving amino acid metabolism (including alanine, aspartate, and glutamate metabolism, tryptophan metabolism, arginine biosynthesis, and arginine and proline metabolism), energy metabolism (encompassing the citrate cycle (TCA cycle), glyoxylate and dicarboxylate metabolism, and butanoate metabolism), and lipid metabolism (particularly sphingolipid metabolism). Notably, PRG-treated cows showed increased concentrations of glutamate, proline, and arginine, which are glucogenic amino acids that can lead to the synthesis of α-ketoglutarate [30]. These amino acids are all closely linked to glutamate production [31]. Specifically, proline and arginine are interconnected within the metabolic pathway of arginine and proline metabolism. Previous studies have reported a progressive decrease in serum proline concentration in ketotic cows [8,30]. Proline and its derivatives are essential components of collagen biosynthesis, contributing to the structure, strength, and integrity of tissues [32]. Additionally, proline has been found to act as a weak agonist of glycine receptors as well as N-methyl-D-aspartate (NMDA) and non-NMDA ionotropic glutamate receptors. Arginine, an important amino acid in the urea cycle, is involved in nerve signal transduction [33], and its reduction is associated with ketosis [34]. In this study, the increased concentrations of these metabolites during the glucogenic process suggest improvements in the urea cycle, signal transduction, and recovery from inflammatory processes in hyperketonemic cows.

Alanine and serine are key glucogenic amino acids involved in pyruvate synthesis [30]. In our study, PRG drenching significantly increased the concentrations of alanine, serine, and pyruvate. In contrast, Lisuzzo et al. [30] found that these metabolites progressively decreased with increasing serum BHBA levels. Notably, serine plays a crucial role as a regulator in glutathione synthesis and is essential for managing oxidative stress [34]. Therefore, the increased serine levels may indicate an enhanced ability of PRG-treated cows to cope with potential oxidative stress. Furthermore, pyruvate and its precursors, alanine, and serine, showed an inverse relationship with BHBA concentration [30], suggesting opposing roles in metabolic regulation. Alanine, being one of the primary resources for gluconeogenesis [35], directly impacts the gluconeogenesis process through its concentration changes. Overall, our results suggest that pyruvate plays a significant role in gluconeogenesis, with its concentration increasing due to the enhanced availability of various substrates in PRG-treated cows.

Tryptophan is an essential amino acid and tryptophan metabolism plays a crucial role in the regulation of immune function, inflammation, and calcium metabolism [36]. There are three main routes of tryptophan catabolism: (1) indole aryl hydrocarbon receptor, (2) kynurenine, and (3) serotonin (5-hydroxytryptamine) pathways [36]. Huang et al. [37] reported that cows with ketosis had a greater plasma concentration of 5-hydroxytryptophan and lower abundance of tryptophan. In the present study, PRG drenching decreased the concentration of 5-hydroxytryptophan and increased tryptophan, indicating that targeting the tryptophan metabolism pathway may hold therapeutic potential for ketosis.

The administration of PRG significantly reduced the relative concentration of kynurenine in cow serum. Kynurenine, a metabolite of tryptophan, can be produced endogenously or by gut microbes [38]. In mammals, most ingested tryptophan is metabolized through the kynurenine pathway [38]. Under normal conditions, kynurenine does not accumulate, but serves as an intermediate in the biosynthesis of the essential cofactor NAD+ from L-tryptophan [39]. However, kynurenine and some of its downstream metabolites, such as DOBA and quinolinic acid, are neurotoxic and have been implicated in cancer progression by suppressing antitumor immune responses and promoting tumor cell survival and motility [40]. Therefore, the accumulation of kynurenine is considered detrimental. Xiong et al. [41] reported that kynurenine is a major metabolic substrate for human glioma through arterial–venous analysis. Additionally, studies have shown decreased levels of kynurenine in the urine of patients responding positively to inflammatory bowel disease treatment [42]. Taken together, the reduction in plasma kynurenine concentration suggests that the health status of ketotic dairy cows administered PRG may be improved.

In the serum of cows administered with PRG, we also observed decreased concentrations of citrate and pyruvate, as well as increased concentrations of 4-aminobutanoate, 2-oxoglutarate, and succinate. These changes are closely related to the metabolism of alanine, aspartate, and glutamate. Notably, these metabolites play significant roles in butanoate metabolism and the tricarboxylic acid (TCA) cycle. Their changes indicate that amino acid metabolism acts as a critical intermediary regulator between lipid and carbohydrate metabolism [43]. The TCA cycle is essential for energy metabolism in animals and, therefore, alterations in its functionality may have certain effects on other metabolic pathways and on the animal itself [44]. Citrate is an intermediate metabolite of TCA, and its concentration was found to be reduced in the milk of ketotic dairy cows, identifying a ketosis-related alteration in energy metabolism [27]. Carnitine is a metabolite responsible for the transport of fatty acids from the cytosol to the mitochondrial matrix, thereby affecting energy metabolism. In blood, carnitine has been identified as being reduced 4 weeks postpartum in animals with disease (metritis, mastitis, laminitis, or retained placenta) during the transition period [45]. Moreover, TCA is functionally related to the urea cycle [30]. In this study, increased levels of citrate and carnitine were observed in PRG cows. Our findings may suggest a possible modulation of the urea cycle and synthesis and improvement of energy metabolism.

In the present study, we also found that sphingolipid metabolism was altered by RPG drenching. Recently, we observed heightened concentrations of lipids associated with sphingolipid metabolism as the severity of fat mobilization increased [15]. Ketotic cows had greater contents of ceramides in the serum and adipose samples compared with healthy cows [14]. In this study, the serum ceramide species concentration in ketotic dairy cows administrated with PRG significantly decreased and was correlated with serum biochemical parameters, such as NEFA and GSH-Px. This indicates that PRG may alleviate metabolic disorders associated with ketosis in dairy cows by affecting sphingolipid metabolism. After administering PRG, we observed a significant reduction in serum NEFA concentrations, indicating inhibited lipolysis in adipose tissue. Ceramides are primarily synthesized de novo from saturated fatty acids, especially palmitate, within the endoplasmic reticulum. Therefore, the decreased NEFA levels may redirect their flow towards the de novo synthesis pathway, potentially leading to lower contents of ceramides in the blood.

Ceramides, a class of sphingolipids, function as signaling molecules to suppress cell proliferation, facilitate apoptosis, and modulate inflammation [46]. Ceramide is a potential antagonist of insulin-stimulated glucose utilization by adipose tissue and skeletal muscle tissue in dairy cows [47]. Rico et al. [48] evaluated plasma concentrations of ceramides in periparturient dairy cows as potential biomarkers of insulin resistance and found that specific ceramides (e.g., C24:0) were positively correlated with NEFA concentrations and negatively correlated with insulin sensitivity. In our study, we observed that the anti-inflammatory cytokine IL-10 concentration was negatively correlated with ceramide species (i.e., Cer (d16:0/16:0), Cer (d12:0/18:0).

A significant correlation between the anti-inflammatory cytokine IL-10 and ceramides has also been identified. Multiple studies have increasingly highlighted the close relationship between sphingolipids and inflammatory responses [49]. Specifically, inflammatory cytokines such as TNF-α in serum and adipose tissue can stimulate ceramide production via TLR4-dependent and TNF-α-dependent pathways. Additionally, research has shown that NEFA can trigger inflammasome activation in macrophages, suggesting that lipotoxic intermediates like ceramides may play a crucial role in inflammasome activation [50].

Furthermore, our observations indicate that drenching with PRG can elevate serum levels of GSH-Px and SOD, suggesting that PRG may have the potential to alleviate oxidative stress in dairy cows. The relationship between ceramides and oxidative stress is well recognized in non-ruminants [51]. On the one hand, although ceramides are essential for maintaining normal mitochondrial function, excessive levels of mitochondrial ceramides can lead to mitochondrial dysfunction. This can promote the generation of ROS, thereby increasing oxidative stress, reducing ATP production, disrupting the electron transport chain, potentially causing apoptosis, and altering the permeability of the mitochondrial outer membrane [52]. On the other hand, H2O2, an inflammatory oxidant, and hypoxia, which causes irreversible damage to multiple cellular systems, have been used to demonstrate that oxidant-induced apoptosis is mediated through the SM-ceramide pathway [53]. In fact, ROS can stimulate the production of ceramides, which, in turn, may inhibit the function of isolated mitochondrial electron transport at complex III, further increasing reactive oxygen species [54]. Therefore, drenching with propylene glycol may alleviate excessive lipolysis in the adipose tissue of ketotic dairy cows, reducing NEFA concentrations, which, in turn, decreases ceramide synthesis and mitigates systemic oxidative stress.

These findings suggest that PRG drenching could be a valuable strategy for dairy farmers in managing ketosis, potentially improving animal health and milk production. Incorporating PRG into treatment protocols might enhance herd productivity and reduce economic losses associated with metabolic disorders. Furthermore, the insights gained from this study could inform the development of more targeted nutritional and management strategies aimed at preventing ketosis and promoting metabolic health in dairy herds. The potential limitations of our study primarily include the sample selection method and the limited sample size used for the untargeted metabolomics and lipidomics analyses. We were unable to collect serum samples during the treatment period, which somewhat limited the comprehensiveness of the study. Although it is intriguing to speculate on the biological roles of these differentially abundant metabolites, confirming their specific roles in host metabolism is beyond the scope of this study and will require further validation in subsequent independent experiments. Additionally, increasing the number of biological replicates could enhance the robustness of our OPLS-DA models. Therefore, we recommend that future studies with similar designs in untargeted metabolomics should consider increasing the number of biological replicates. It is also important to emphasize that our data-driven approach based on untargeted metabolomics is intended to generate new, testable hypotheses for future research.

## 5. Conclusions

In summary, this study demonstrates that PRG drenching effectively alleviates oxidative stress and enhances immune function in ketotic cows by modulating amino acid and lipid metabolism. The administration of PRG resulted in significant reductions in serum NEFA, BHBA, and CRP levels, and increases in glucose, anti-inflammatory factor IL-10, SOD, and GSH-Px activities. Metabolomic and lipidomic analyses revealed alterations in multiple metabolic pathways, particularly those involving glucogenic amino acids and sphingolipid metabolism. These findings indicate that PRG can restore metabolic homeostasis and mitigate the adverse effects of ketosis.

These findings advance the field by offering a deeper understanding of PRG‘s multifaceted role in improving metabolic health in ketotic cows. The study provides evidence for the potential use of PRG as a therapeutic intervention, which can be integrated into management practices for improving the health and productivity of dairy cows affected by ketosis. For future research, studies with larger sample sizes and the application of targeted metabolomics and lipidomics are recommended to validate these findings and further elucidate the mechanisms by which PRG affects ketosis. Such studies could help refine the understanding of PRG’s impact on specific metabolic pathways and improve treatment strategies for metabolic disorders in dairy cows.

## Figures and Tables

**Table 1:** Effects of propylene glycol drenching on serum glucose and lipid metabolism biomarkers of ketotic cows.

Item ^1^	Treatment ^2^		SEM	*p*-Value
	CON	PRG		
NEFA, mmol/L	0.673	0.428	0.0513	0.005
BHBA, mmol/L	1.259	0.914	0.0843	0.010
Glucose, mg/dL	47.34	53.82	2.016	0.019
Insulin, uU/mL	14.10	14.65	0.484	0.309
TG, mmol/L	0.071	0.056	0.0113	0.257
Leptin, μg/L	4.40	3.83	0.226	0.048
Adiponectin, mg/L	43.75	38.63	3.804	0.236
RQUICKI	0.377	0.396	0.003	0.004

^1^ Blood sampled by puncture of coccygeal vessels from cows after three consecutive days of propylene glycol drenching. NEFA, non-esterified fatty acid; BHBA, β-hydroxybutyric acid; TG, triglyceride; RQUICKI, revised quantitative insulin sensitivity check index. ^2^ CON, ketosis cows without treatment, PRG, ketosis cows with propylene glycol drenching.

**Table 2:** Effects of propylene glycol drenching on serum antioxidant and inflammation indicators in ketotic cows.

Item ^1^	Treatment ^2^		SEM	*p*-Value
	CON	PRG		
SOD, mmol/L	60.14	77.77	2.935	0.004
GSH-Px, mmol/L	258.14	373.98	11.123	<0.001
LPS, EU/mL	0.50	0.31	0.038	0.019
CRP, ng/L	17.68	10.95	1.020	0.028
IL-1β, ng/L	73.25	76.62	5.94	0.595
IL-2, ng/L	236.44	259.93	11.704	0.101
IL-6, ng/L	16.80	13.18	1.793	0.098
IL-10, μg/L	3.83	4.40	0.226	0.048
TNF-α, ng/L	265.39	259.48	19.658	0.776

^1^ Blood sampled by puncture of coccygeal vessels from cows after three consecutive days of propylene glycol drenching. SOD, superoxide dismutase; GSH-Px, glutathione peroxidase; LPS, lipopolysaccharide; MDA, malonaldehyde; CRP, C-reactive protein; IL, interleukin; TNF-α, tumor necrosis factor α. ^2^ CON, ketosis cows without treatment, PRG, ketosis cows with propylene glycol drenching.

## Data Availability

The datasets used and/or analyzed during the current study are available from the corresponding author on reasonable request.

## Supplementary Materials

The following supporting information can be downloaded at https://www.mdpi.com/article/10.3390/antiox13091146/s1, Table S1: Ingredients and chemical composition of the diet; Table S2: Basic description of used cows in the study; Table S3: Serum glucose and lipid metabolism biomarkers of ketotic cows on the day before propylene glycol administration; Table S4: Serum antioxidant and inflammation indicators of ketotic cows on the day before propylene glycol administration; Table S5: Differential serum metabolite in between CON and PRG cows; Table S6: Differential lipid metabolite in between CON and PRG cows.
