# Supplementary material for: Propylene Glycol Alleviates Oxidative Stress and Enhances Immunity in Ketotic Cows through Modulating Amino Acid and Lipid Metabolism
Source: Antioxidants (Basel). 2024 Sep 23;13(9):1146. doi: 10.3390/antiox13091146 (PMC11428896; doi:10.3390/antiox13091146)
Supplement: Supplementary file 1 [file antioxidants-13-01146-s001.zip › antioxidants-3169619-supplementary.pdf]

Table S1. Ingredients and chemical composition of the diet.

Item[1]	Prepartum	Early lactation
Ingredient, % of DM		
Corn silage	25.7	30.4
Alfalfa silage	-	7.84
Oat Hay	45.6	-
Alfalfa hay	-	10.4
Soybean meal	7.68	16.2
Soybean hull	7.74	3.65
Crushed corn	3.88	9.46
Corn steam flakes	-	9.30
Whole cottonseed	-	5.36
Corn gluten meal	4.08	-
Sugarcane molasses	-	2.59
DCAD supplement	1.01	1.01
Mineral and vitamin mix[2]	4.31	3.70
Chemical composition, % of DM		
DM, as fed %	51.2	47.8
CP	15.6	17.5
NFC[3]	33.2	38.5
NDF	43.5	33.2
ADF	23.5	19.3
EE	3.48	5.10
Ash	5.10	5.68
NEL (Mcal/kg of DM)	1.38	1.69
DCAD[4]	−98.1	291

[1]DM = dry matter; DCAD = dietary cation-anion difference; CP = crude protein; NFC = non-fiber carbohydrates; NDF = neutral detergent fiber; ADF = acid detergent fiber; EE = ether extract; NEL = net energy for lactation.

[2]Prepartum premix: 1 kg of premix included 1,400,000 IU Vitamin A, 437,500 IU Vitamin D, 19,000 IU Vitamin E, 2,000 mg Cu, 3,600 mg Mn, 9,000 mg Zn, 120 mg Se, 160 mg I, 100 mg Co. Early lactation premix: 1 kg of premix included 150,000 IU Vitamin A, 35,000 IU Vitamin D, 2,000 IU Vitamin E, 250 mg Cu, 500 mg Mn, 1,000 mg Zn, 20 mg Se, 40 mg I, 25 mg Co.

[3]NFC = DM − (ash + CP + EE + NDF).

[4]DCAD (mEq/kg of DM) = (Na + K) − (Cl + S), where the unit of Na, K, Cl, and S is mEq/kg of DM.

Table S2. Basic description of used cows in the study[1]

Item[2]	CON[1] (n = 6)	PRG[2] (n = 6)	*P*-value
Parity	2.7 ± 0.3	2.8 ± 0.4	0.831
BW (kg)	640.48 ± 8.01	633.86 ±10.03	0.302
BCS	3.60 ± 0.05	3.54 ± 0.06	0.422
Milk yield (kg)	28.90 ± 0.97	30.66 ± 1.32	0.219
DMI (kg)	18.46 ± 0.34	17.62 ± 0.77	0.201
BHBA (mmol/L)	1.73 ± 0.15	1.86 ± 0.33	0.452
DIM (d)	10.4 ± 0.52	9.8 ± 0.61	0.615

[1] The data were collected on the day of ketosis diagnosis in dairy cows and are expressed as mean ± standard error of the mean.

[2]BW = bodyweight; BCS = body condition score; DMI = dry matter intake; DIM = days in milk; BHB = β-hydroxybutyric acid.

[3]CON = ketosis cows without treatment; PRG = ketosis cows with propylene glycol drenching.

Table S3. Serum glucose and lipid metabolism biomarkers of ketotic cows on the day before propylene glycol administration.

Item[1]	Treatment[2]		SEM	P-value
	CON	PRG		
NEFA, mmol/L	1.290	1.168	0.141	0.523
BHBA, mmol/L	1.731	1.862	0.196	0.452
Glucose, mg/dL	46.09	52.84	2.373	0.340
Insulin, uU/mL	13.20	12.09	0.240	0.241
TG, mmol/L	0.042	0.039	0.028	0.468
Leptin, µg/L	4.83	4.99	0.026	0.606
Adiponectin, mg/L	38.41	40.92	1.891	0.293
RQUICKI	0.410	0.396	0.004	0.353

[1]Blood sampled by puncture of coccygeal vessels from cows on the day before propylene glycol administration (i.e., the day of ketosis diagnosis). NEFA, non-esterified fatty acid; BHBA, β-hydroxybutyric acid; TG, triglyceride; RQUICKI, revised quantitative insulin sensitivity check index.

[2]CON, ketosis cows without treatment, PRG, ketosis cows with propylene glycol drenching.

Table S4. Serum antioxidant and inflammation indicators of ketotic cows on the day before propylene glycol administration.

Item[1]	Treatment[2]		SEM	P-value
	CON	PRG		
SOD, mmol/L	48.92	46.09	2.935	0.441
GSH-Px, mmol/L	256.18	247.40	13.041	0.592
LPS, EU/mL	0.58	0.52	0.067	0.203
CRP, ng/L	18.70	19.63	1.54	0.721
IL-1β, ng/L	74.33	77.92	3.40	0.655
IL-2, ng/L	256.49	243.21	10.972	0.197
IL-6, ng/L	15.03	16.67	1.302	0.273
IL-10, μg/L	4.94	4.67	0.413	0.306
TNF-α, ng/L	273.94	260.03	12.921	0.620

[1]Blood sampled by puncture of coccygeal vessels from cows on the day before propylene glycol administration (i.e., the day of ketosis diagnosis). SOD, superoxide dismutase; GSH-Px, glutathione peroxidase; LPS, lipopolysaccharide; MDA, malonaldehyde; CRP, C-reactive protein; IL, interleukin; TNF-α, tumor necrosis factor α.

[2]CON, ketosis cows without treatment, PRG, ketosis cows with propylene glycol drenching.

Table S5. Differential serum metabolite in between CON and PRG cows.

Metabolites	VIP	P-value	Log2FC(PRG/CON)	Regulate
Glutamate	4.2016	0.018	0.2779	up
Taurodeoxycholic acid	3.385	0.035	0.1779	up
Butyryl-L-carnitine	2.7296	0.016	0.087	up
Proline	2.6886	0.029	0.0979	up
L-Carnitine	2.6442	0.045	0.0989	up
Aspartate	2.532	0.029	0.0859	up
N-Docosahexaenoyl Threonine	2.2169	0.017	0.0673	up
O-Acetylcarnitine	1.9631	0.032	0.0441	up
DL-Acetylcarnitine	1.8386	0.004	0.0417	up
Serine	1.838	0.041	0.0379	up
Alanine	1.7971	0.019	0.0465	up
Pyruvate	1.4988	0.009	0.025	up
Tryptophan	1.3514	0.035	0.0313	up
Citrate	1.1878	0.003	0.0183	up
Kynurenine	3.975	0.022	-0.2256	down
Gly-Ile	2.916	0.018	-0.1298	down
Glycyl-leucine	2.8777	0.018	-0.1243	down
4-Chlorobenzoic Acid	2.4786	0.025	-0.0818	down
Neuropeptide EI rat	2.1506	0.019	-0.0535	down
Flumethasone	2.1461	0.022	-0.0776	down
Glycyl-Phenylalanine	2.1167	0.009	-0.0645	down
15H-11,12-EETA	2.1146	0.034	-0.0639	down
L-Palmitoylcarnitine	1.8453	0.042	-0.0452	down
Ethyl benzoate	1.8104	0.032	-0.0391	down
Hexadecanedioic acid	1.7732	0.027	-0.0394	down
Palmitoleic acid	1.7197	0.027	-0.0396	down
5-Hydroxy-L-tryptophan	1.6414	0.028	-0.0397	down
4-Aminobutanoate	1.6028	0.028	-0.0393	down
9,12,13-TriHOME	1.489	0.048	-0.0292	down
2,3-dinor Prostaglandin E1	1.4433	0.033	-0.0289	down
13(S)-HpODE	1.3454	0.028	-0.0224	down
2-oxoglutarate	1.3248	0.033	-0.0228	down
Fusaric Acid	1.3028	0.033	-0.0198	down
Vanillylmandelic acid	1.2895	0.003	-0.0211	down
Umbelliferone	1.1594	0.009	-0.0161	down
Norvaline	1.0993	0.012	-0.0156	down
Succinate	1.0353	0.018	-0.0139	down

VIP = variable importance in the projection; FC= fold change; CON = ketosis cows without treatment; PRG = ketosis cows with propylene glycol drenching.

Table S6. Differential lipid metabolite in between CON and PRG cows.

Metabolites	VIP	P-value	FC(PRG/CON)	Regulate
cPA(18:2)	3.5408	0.030	1.1803	up
cPA(16:0)	2.6592	0.030	1.0923	up
PC(18:3/18:2)	2.1914	0.014	1.0451	up
LdMePE(18:2)	2.0555	0.006	1.0463	up
MLCL(14:2/20:3/20:3)	2.0253	0.004	1.0401	up
MLCL(14:2/18:2/18:2)	2.0228	0.006	1.0364	up
LPC(20:3)	2.0026	0.000	1.0497	up
MLCL(14:2/14:0/14:0)	2.0003	0.004	1.0424	up
PC(20:5/18:2)	1.9782	0.013	1.0419	up
LPC(18:4)	1.9399	0.002	1.0561	up
MGDG(14:0e/12:2)	1.9112	0.037	1.0463	up
MLCL(10:2/16:1/20:1)	1.9101	0.002	1.0339	up
LPC(18:2)	1.9055	0.003	1.0416	up
SM(d16:1/18:3)	1.8808	0.000	1.0459	up
SM(t18:0/23:0)	1.8759	0.001	1.0479	up
PC(16:1/18:2)	1.8372	0.023	1.0324	up
PC(20:4e/13:0)	1.8218	0.005	1.0453	up
SM(d19:0/24:5)	1.8153	0.000	1.0422	up
LPC(18:3)	1.8047	0.009	1.047	up
PC(16:1/22:6)	1.7995	0.009	1.0497	up
LPC(14:0)	1.7933	0.002	1.0453	up
PC(18:3e/13:0)	1.7905	0.009	1.0475	up
PC(10:0e/22:6)	1.7779	0.019	1.0612	up
LPC(20:2)	1.7777	0.004	1.0485	up
LPC(16:1)	1.7712	0.003	1.0405	up
LPC(16:2e)	1.7217	0.008	1.0452	up
SM(t18:0/24:0)	1.6829	0.002	1.0402	up
PC(15:0/24:2)	1.6732	0.002	1.0443	up
PC(16:0/24:2)	1.673	0.000	1.0412	up
PC(17:0/24:2)	1.672	0.006	1.0481	up
LPC(18:1)	1.664	0.019	1.0395	up
LPC(20:5)	1.6581	0.018	1.0451	up
PS(16:0e/18:2)	1.658	0.025	1.0341	up
SM(d18:2/18:3)	1.6271	0.005	1.0379	up
PI(17:0/18:2)	1.6255	0.035	1.0281	up
SM(d18:2/14:0)	1.6223	0.023	1.0485	up
SM(d16:1/12:0)	1.5722	0.022	1.0445	up
SM(t18:0/22:0)	1.5661	0.003	1.0356	up
PC(18:2/18:2)	1.5484	0.025	1.0338	up
MePC(15:0/20:4)	1.5484	0.025	1.0338	up
PC(20:0/18:2)	1.5253	0.000	1.0298	up
MLCL(14:2/18:1/18:1)	1.5215	0.014	1.0229	up

PC(16:0/22:0)	1.5128	0.030	1.0372	up
SM(t18:0/16:0)	1.5125	0.003	1.0326	up
PC(18:0/20:2)	1.5044	0.023	1.0339	up
MLCL(14:2/16:0/16:0)	1.4986	0.008	1.0176	up
SM(d18:1/24:0)	1.4959	0.005	1.0285	up
SM(d20:0/24:5)	1.491	0.001	1.0299	up
SM(d17:1/18:1)	1.4857	0.020	1.0359	up
SM(d18:2/24:3)	1.4744	0.018	1.0347	up
PC(15:0/18:3)	1.4696	0.035	1.0397	up
SM(d18:2/16:1)	1.4662	0.016	1.0377	up
PC(12:0e/20:4)	1.4629	0.016	1.0362	up
SM(d19:1/24:0)	1.4591	0.006	1.029	up
PC(12:1e/18:2)	1.4516	0.036	1.0418	up
PC(18:3e/15:0)	1.4454	0.009	1.0253	up
LdMePE(16:0)	1.4353	0.012	1.019	up
LPC(16:1e)	1.4213	0.007	1.0277	up
LPC(19:0)	1.4088	0.016	1.0325	up
Hex1Cer(t18:0/24:0+O)	1.3912	0.001	1.0309	up
SM(d19:0/24:4)	1.3842	0.020	1.0258	up
LPC(15:0)	1.3831	0.044	1.0329	up
SM(d14:0/15:1)	1.3821	0.008	1.0344	up
PC(8:0e/9:0)	1.3752	0.047	1.0372	up
SM(t18:1/17:1)	1.375	0.041	1.0328	up
SM(d16:1/23:0)	1.3737	0.004	1.0225	up
PC(8:1e/21:0)	1.3727	0.016	1.0284	up
LPC(22:0)	1.3677	0.026	1.0351	up
PC(19:0/18:2)	1.3638	0.003	1.0239	up
SM(d18:1/12:0)	1.3431	0.017	1.0298	up
SM(d18:1/13:0)	1.338	0.004	1.0274	up
SM(d16:1/16:0)	1.3345	0.004	1.0216	up
LPC(16:0e)	1.329	0.002	1.0242	up
LdMePE(18:1)	1.3255	0.031	1.0233	up
Hex1Cer(t18:0/23:0+O)	1.3221	0.002	1.0265	up
PC(16:2e/12:0)	1.3171	0.045	1.0311	up
LPC(16:0)	1.3015	0.009	1.0189	up
SM(d18:0/21:0)	1.2964	0.021	1.0258	up
SM(d18:2/23:0)	1.2885	0.017	1.0229	up
PC(18:0/24:2)	1.288	0.001	1.0248	up
PC(16:0/21:1)	1.2765	0.039	1.0262	up
PI(18:0/16:0)	1.2537	0.048	1.0243	up
LPC(34:0)	1.2348	0.026	1.0265	up
SM(d18:2/16:0)	1.2307	0.006	1.0194	up
SM(d18:1/23:0)	1.2262	0.031	1.0227	up
PC(16:0/16:1)	1.2234	0.003	1.0168	up

PC(20:0e/18:2)	1.2178	0.031	1.0259	up
LdMePE(18:0)	1.1528	0.013	1.0131	up
SM(d16:1/24:3)	1.145	0.007	1.0185	up
SM(t18:1/16:0)	1.141	0.046	1.0226	up
SM(d18:1/21:1)	1.1327	0.021	1.0204	up
PC(19:1/18:2)	1.1103	0.022	1.0174	up
SM(d16:1/22:0)	1.1016	0.006	1.0155	up
SM(d17:1/16:0)	1.0922	0.037	1.017	up
SM(d14:0/18:0)	1.0704	0.043	1.0188	up
PC(16:1/18:3)	1.0692	0.030	1.0198	up
SM(d18:1/22:2)	1.0349	0.023	1.0165	up
PC(16:1/20:5)	1.0313	0.043	1.0168	up
PC(18:0e/20:3)	1.0189	0.028	1.0152	up
MLCL(14:2/18:0/18:0)	1.0025	0.023	1.0087	up
SM(d18:2/16:0)	3.5408	0.003	1.1803	up
SM(d18:1/23:0)	2.6592	0.003	1.0923	up
PC(16:0/16:1)	2.1914	0.005	1.0451	up
PC(20:0e/18:2)	2.0555	0.002	1.0463	up
LdMePE(18:0)	2.0253	0.010	1.0401	up
SM(d16:1/24:3)	2.0228	0.000	1.0364	up
SM(t18:1/16:0)	2.0026	0.000	1.0497	up
SM(d18:1/21:1)	2.0003	0.006	1.0424	up
PC(19:1/18:2)	1.9782	0.018	1.0419	up
SM(d16:1/22:0)	1.9399	0.018	1.0561	up
SM(d17:1/16:0)	1.9112	0.001	1.0463	up
SM(d14:0/18:0)	1.9101	0.001	1.0339	up
PC(16:1/18:3)	1.9055	0.003	1.0416	up
SM(d18:1/22:2)	1.8808	0.042	1.0459	up
PC(16:1/20:5)	1.8759	0.006	1.0479	up
PC(18:0e/20:3)	1.8372	0.032	1.0324	up
MLCL(14:2/18:0/18:0)	1.8218	0.004	1.0453	up
SM(d18:2/16:0)	1.8153	0.001	1.0422	up
SM(d18:1/23:0)	1.8047	0.037	1.047	up
PC(16:0/16:1)	1.7995	0.001	1.0497	up
PC(20:0e/18:2)	1.7933	0.001	1.0453	up
LdMePE(18:0)	1.7905	0.005	1.0475	up
SM(d16:1/24:3)	1.7779	0.001	1.0612	up
SM(t18:1/16:0)	1.7777	0.007	1.0485	up
SM(d18:1/21:1)	1.7712	0.001	1.0405	up
PC(19:1/18:2)	1.7217	0.003	1.0452	up
SM(d16:1/22:0)	1.6829	0.016	1.0402	up
SM(d17:1/16:0)	1.6732	0.000	1.0443	up
SM(d14:0/18:0)	1.673	0.033	1.0412	up
PC(16:1/18:3)	1.672	0.006	1.0481	up

SM(d18:1/22:2)	1.664	0.001	1.0395	up
PC(16:1/20:5)	1.6581	0.010	1.0451	up
PC(18:0e/20:3)	1.658	0.001	1.0341	up
MLCL(14:2/18:0/18:0)	1.6271	0.001	1.0379	up
OAHFA(18:1/18:0)	2.6191	0.003	0.9392	down
OAHFA(18:1/20:3)	2.4089	0.003	0.943	down
OAHFA(18:0/20:2)	2.2566	0.005	0.9445	down
OAHFA(16:0/18:2)	2.1349	0.002	0.9503	down
Hex1Cer(d18:1/16:0)	2.0923	0.010	0.9447	down
Cer(d22:1/16:0)	2.0215	0.000	0.9459	down
Cer(d20:0/16:0)	1.8561	0.000	0.9592	down
PE(18:1/20:4)	1.8414	0.006	0.9629	down
MGDG(14:0e/16:0)	1.8115	0.018	0.957	down
PE(18:0/20:4)	1.7737	0.018	0.9739	down
Cer(d20:0/18:0)	1.693	0.001	0.965	down
TG(18:1/18:1/18:1)	1.6738	0.001	0.9638	down
SPH(d22:2)	1.6462	0.003	0.9591	down
dMePE(16:0/22:5)	1.6425	0.042	0.9678	down
CL(20:4/16:1/18:0/24:1)	1.6412	0.006	0.9625	down
MGDG(16:0e/18:0)	1.6368	0.032	0.961	down
TG(18:0/18:1/20:4)	1.6275	0.004	0.963	down
MePC(18:4/16:1)	1.6174	0.001	0.9694	down
Hex1Cer(d16:0/26:1)	1.5809	0.037	0.9745	down
TG(18:2e/9:0/9:0)	1.5513	0.001	0.9705	down
DG(18:1/18:1)	1.5513	0.001	0.9705	down
TG(18:0/18:1/18:3)	1.5214	0.005	0.9684	down
Cer(d12:0/18:0)	1.5197	0.001	0.9703	down
TG(17:0/18:1/18:1)	1.5173	0.007	0.9673	down
Cer(d16:0/16:0)	1.5158	0.001	0.9734	down
Cer(t18:0/18:0)	1.504	0.003	0.9669	down
TG(16:1e/9:0/9:0)	1.4945	0.016	0.966	down
Cer(d16:0/18:0+O)	1.4934	0.000	0.9705	down
TG(18:0/16:0/20:4)	1.4912	0.033	0.9668	down
TG(9:0/9:0/18:1)	1.4837	0.006	0.9678	down
Cer(d14:0/16:0)	1.469	0.001	0.9728	down
MePC(15:0/18:2)	1.4678	0.010	0.9722	down
TG(18:4/16:0/18:1)	1.4618	0.001	0.973	down
Cer(d12:0/16:0)	1.4477	0.001	0.9738	down
ChE(16:0)	1.4223	0.010	0.9718	down
MGDG(14:0e/18:1)	1.3981	0.016	0.983	down
Cer(d18:0/16:0)	1.3763	0.008	0.9741	down
MePC(18:4/20:4)	1.3742	0.000	0.9786	down
TG(16:0/14:1/18:3)	1.3266	0.005	0.9749	down
SPH(d20:1)	1.3255	0.001	0.9767	down

SM(d18:1/20:3)	1.3252	0.003	0.9772	down
TG(16:0/16:0/20:5)	1.3135	0.022	0.9753	down
TG(16:0/18:1/18:1)	1.3101	0.015	0.9746	down
CL(18:2/20:3/20:0/24:1)	1.301	0.004	0.9776	down
TG(16:0/14:0/18:1)	1.2994	0.003	0.9798	down
PC(14:0e/16:0)	1.2981	0.044	0.9786	down
Cer(d22:0/18:0)	1.2874	0.007	0.9775	down
CerPE(d16:0/17:0)	1.2739	0.020	0.9738	down
TG(16:0/14:0/18:3)	1.2688	0.004	0.9767	down
TG(16:1/16:1/18:1)	1.2636	0.005	0.9795	down
SPH(t16:1)	1.2626	0.002	0.9754	down
SPH(d18:1)	1.2466	0.005	0.9782	down
CL(20:4/16:0/20:0/22:0)	1.2305	0.008	0.9783	down
DG(18:1e/18:1)	1.2138	0.012	0.9782	down
SPH(d16:1)	1.2085	0.001	0.9816	down
SM(d18:0/16:0)	1.2036	0.010	0.9811	down
PI(18:0/20:4)	1.1975	0.036	0.9871	down
TG(16:1/14:0/20:4)	1.1694	0.014	0.9805	down
PC(18:0/20:4)	1.1536	0.026	0.9888	down
PC(16:0/20:4)	1.1512	0.045	0.9869	down
SPH(t18:0)	1.1436	0.035	0.9803	down
TG(16:1/16:1/16:1)	1.1375	0.046	0.9796	down
LSM(t20:0)	1.1194	0.014	0.9811	down
TG(16:1/14:0/14:0)	1.1076	0.035	0.9799	down
TG(16:0/16:0/18:1)	1.0962	0.028	0.9839	down
TG(16:0/14:0/14:0)	1.0896	0.035	0.9805	down
TG(16:0/14:0/16:1)	1.0799	0.042	0.9821	down
TG(16:0/18:1/18:3)	1.0787	0.026	0.9821	down
dMePE(16:0/18:1)	1.0777	0.013	0.9848	down
dMePE(16:0/18:2)	1.0623	0.044	0.9836	down
TG(15:0/16:1/16:1)	1.0562	0.000	0.9857	down
PC(18:1/18:1)	1.0553	0.029	0.9899	down
TG(16:0/16:0/16:0)	1.0237	0.013	0.9857	down
TG(16:1/14:0/18:1)	1.0187	0.041	0.9847	down
TG(16:0/18:3/18:3)	1.0116	0.037	0.9829	down

VIP = variable importance in the projection; FC= fold change; CON = ketosis cows without treatment; PRG = ketosis cows with propylene glycol drenching.
